# Stochasticity, Entropy and Neurodegeneration

**DOI:** 10.3390/brainsci12020226

**Published:** 2022-02-07

**Authors:** Peter K. Panegyres

**Affiliations:** 1 Neurodegenerative Disorders Research Pty Ltd., Perth 6005, Australia; research@ndr.org.au; 2 School of Medicine, The University of Western Australia, Perth 6009, Australia

**Keywords:** stochasticity, space-time, entropy, neurodegeneration

## Abstract

We previously suggested that stochastic processes are fundamental in the development of sporadic adult onset neurodegenerative disorders. In this study, we develop a theoretical framework to explain stochastic processes at the protein, DNA and RNA levels. We propose that probability determines random sequencing changes, some of which favor neurodegeneration in particular anatomical spaces, and that more than one protein may be affected simultaneously. The stochastic protein changes happen in three-dimensional space and can be considered to be vectors in a space-time continuum, their trajectories and kinetics modified by physiological variables in the manifold of intra- and extra-cellular space. The molecular velocity of these degenerative proteins must obey the second law of thermodynamics, in which entropy is the driver of the inexorable progression of neurodegeneration in the context of the N-body problem of interacting proteins, time-space manifold of protein-protein interactions in phase space, and compounded by the intrinsic disorder of protein-protein networks. This model helps to elucidate the existence of multiple misfolded proteinopathies in adult sporadic neurodegenerative disorders.

## 1. Introduction

In our previous studies, we postulated that stochastic processes were important in the pathogenesis of sporadic adult onset neurodegenerative disorders [1]. To summarize, random sequence or other changes in proteins, generated at a DNA, RNA or peptide level, provide the kernel that generates, cultivates, and eventually propagates the neurodegenerative process in an appropriate intra- and extracellular milieu. Such considerations might also be relevant to other conditions such as cancer. In this study, we wish to enunciate this emphasizing a theoretical framework that enhances our understanding of the four-dimensional nature of the pathophysiological mechanisms and the important role of entropy as an operator of the neurodegenerative process.

To the best of our knowledge, there are no communications that attempt to provide a conceptual mathematical basis for the origin, propagation, and progression of neurodegeneration [2,3]. A single publication deals with computer simulation of stochastic models of polypeptide amyloid-beta in Alzheimer’s disease [4]. Stochastic mathematical concepts have been outlined to enhance the design of clinical trials in Alzheimer’s disease [5], and to model biological networks [6].

## 2. Materials and Methods

This communication is theoretically based on our previously published investigations [1,7].

## 3. Results and Discussion

It is the author’s suggestion that the probability that neurodegeneration occurs in a particular anatomical space, $P_{AS}\left(neurodeg\right),$ is a function, f, of $P_{DNA}$, $P_{RNA},$ and $P_{PROTEIN}$, where:

$P_{DNA}$ expresses the probability that a DNA sequence will modify to advance neurodegeneration,

$P_{RNA}$ expresses the probability that an RNA sequence will change to enable neurodegeneration,

$P_{PROTEIN}$ connotes the probability that a protein sequence, say the Aβ peptide important in Alzheimer’s disease, will alter its composition to favor neurodegeneration.

It is possible that more than one of these probabilities may simultaneously be non-zero in the same neuron or other cell types, such as glial cells. It is also possible that each of these probabilities may take on different values in different anatomical brain regions, and that different proteins may be affected simultaneously.

Thus:

$$P_{AS}\left(neurodeg\right)=f(P_{DNA},\;P_{RNA},\;P_{PROTEIN})$$

Such a concept helps to comprehend the regional nature of young onset dementia; for example, frontal variants of Alzheimer’s disease versus temporal linguistic forms.

Determining f is the ultimate goal of this stochastic approach. To make any progress, our study needs to mathematically analyze the motion of protein sequences. A protein sequence (PS) will change by stochastic forces and enable neurodegeneration:

$$PS_{PHYSIOL}\;\to \;PS_{NEURODEG}$$

This process occurs in three-dimensional intracellular and extracellular space $\left[R^{3}\right]$.

**Figure 1:**
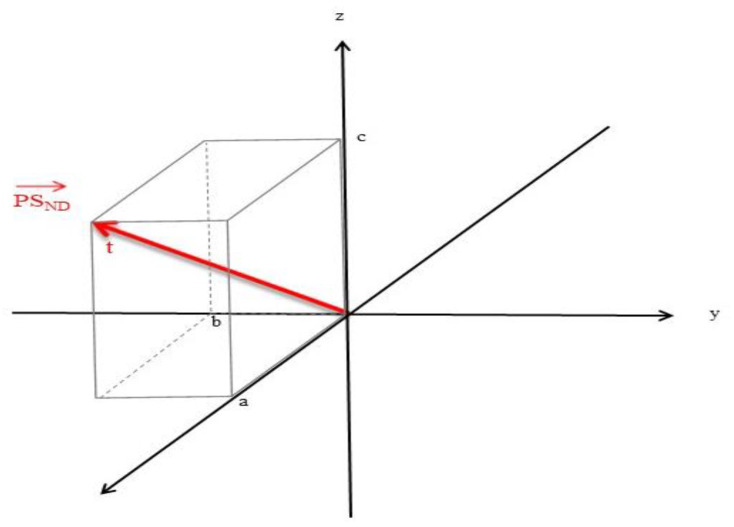
A protein sequence (PS) involved in neurodegeneration represented in three-dimensional space and time.

We will begin by representing a PS as a position vector, $\vec{PS},$ in $R^{3}$. The location of the origin and orientation of the axes is arbitrary for this illustration (Figure 1).

Figure 1 shows the vector broken down into its components, a, b, and c. This position vector is a function of time (t).

The pathway of single aberrant molecule $PS_{ND}$ will drive the production of other $PS_{ND}$ by aberrant biochemical feedback mechanisms at a DNA, RNA or protein level resulting in an overabundance of $PS_{ND}$:

$$PS_{ND}\;\to \;PS_{ND1}+PS_{ND2}+PS_{ND3}+\cdots +PS_{NDx}$$

The production of proteins is tightly regulated to maintain the constancy of the milieu interieur. If we take the example of prion protein production *PrP^C^* → *PrP^SC^*, *PrP^C^* is synthesized and post-translationally modified in the endoplasmic reticulum (ER), transported to the cell membrane after modification in the Golgi body. In the ER, the protein undergoes cleavage of the N– and C–terminal signal peptides, followed by the addition of N–linked glycan molecules at two sites, as well as a glycosylphosphatidylinositol (GPI) anchor, a single disulfide bond is then formed. *PrP^C^* travels to the cell membrane, some *PrP^C^* is internalized into endosomes, most is recycled to the cell membrane; some *PrP^C^* may be released into the extracellular space by cleavage within the GPI anchor. *PrP^C^* → *PrP^SC^* may occur in cell membranes, endosomes or lysosomes [8]. As this system is tightly regulated, we posit that a single aberrant *PrP^SC^*, generated from alpha-helix rich *PrP^C^*, to the β-sheet, is sufficient to disrupt the physiological function of *PrP^C^*, resulting in *PrP^SC^* overproduction → aggregation → insolubility → disruption of the control of *PrP* ssynthesis → overproduction → neurodegeneration, with *PrP^SC^* binding *PrP^C^* resulting in further intracellular disruption, and their abnormal conformations → self-propagation → resulting in disease [9]. Similar considerations apply to the proteins involved in Alzheimer’s disease, Parkinson’s disease, and frontotemporal dementia. Protective mechanisms may prevent this happening to every aberrant molecule.

**Figure 2:**
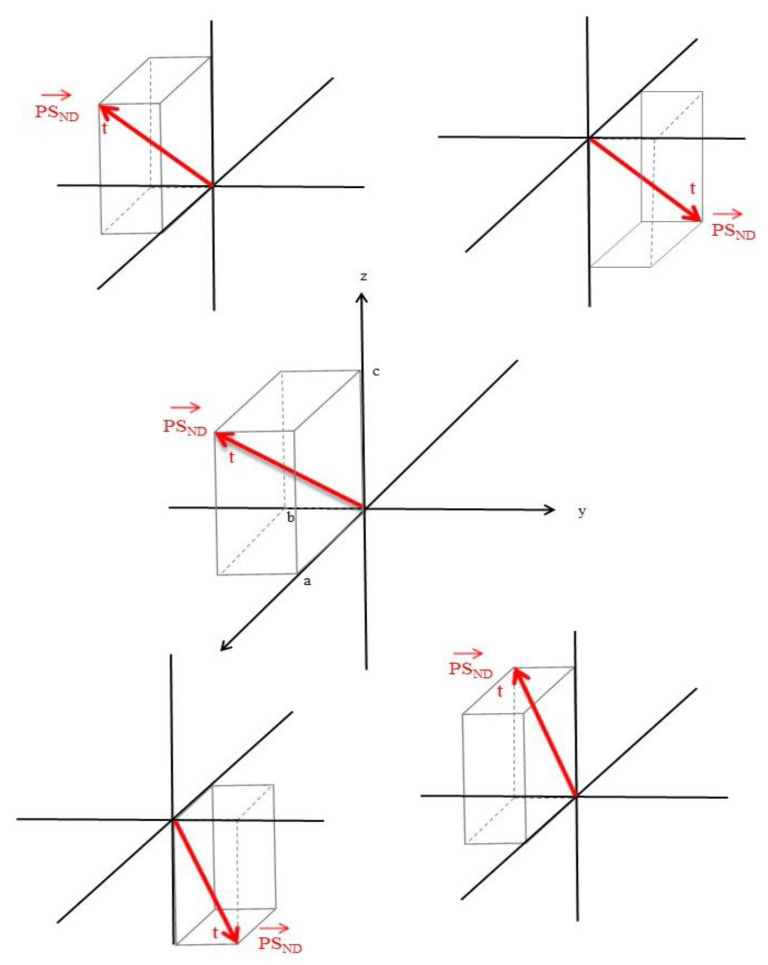
The trajectories of multiple protein sequences (PS) in space and time in neurodegeneration. *PS_ND_* = protein sequence, neurodegeneration.

The passage of our single $PS_{ND}$ molecule will be affected by other $PS_{ND}$ in three-dimensional intracellular and extracellular space, each with its own trajectory (Figure 2).

These three-dimension considerations allow us to factor time, anatomical location, biophysical microenvironmental effects—such as pH—atypical protein folding, degradation mechanisms, over-production and cellular mechanisms, such as immunological reactions, and microglial cellular responses that influence normal protein traffic. Furthermore, as the pathological molecules aggregate both intracellularly and extracellularly the probability increases that collisions between the molecules will occur, compromising $PS_{ND}$ movement and flow, disrupting the physiological kinetics of protein intracellular/extracellular motion and advancing neurodegeneration. In the manifold of intracellular and extracellular space, as the physiological manifold disrupts the dimensions influencing $PS_{ND}\;$motion, the process moves ever forward towards neurodegeneration.

These considerations of the movement of molecules in $R^{3}$ intracellular space raises questions as to the kinetics and molecular velocity distribution of $PS_{ND}$ in cellular and extracellular spaces and is of greater complexity than the concepts developed for kinetic theory of gases, but nevertheless must involve the second law of thermodynamics in which entropy is an intrinsic component of a thermodynamic system—like a cell. Boltzmann developed the H-theorem, in which molecular velocity distribution acts like thermodynamic entropy [10]. Probability and mechanics are important in $PS_{ND}$ action in the manifold of intracellular space and time as developed above.

When systems reach the equilibrium of a normal cell, the interactions between proteins in the cell approach the classical N-body problem, an attempt to predict the movement of one molecule in relation to another. In physiological systems, there are many degrees of freedom and the interactions that determine equilibrium is expressed as:

$$H=\sum^{\;}H_{j}$$

*H* = Hamiltonian = total energy of a system.
*H_j_* = molecular kinetic energies.

This formula applies to states such as gases and solids and has some validity for the complexity of biological systems. The N-body complexity of interacting $PS_{ND}$ may be approached by perturbation theory, which then becomes an extremely difficult problem for the brain cell and its intracellular proteins in dynamic physiological systems and even greater in the pathological state when equilibrium is disrupted. The N–body problem was developed as a means of predicting the individual motions of a group of interacting celestial bodies, where forces such as gravity are operative. In protein and cellular assemblies in structural biology, the Coulomb potential has the same form as the gravitational potential, and the charges may be positive or negative, resulting in repulsive as well as attractive forces which influence molecular interaction and movement in a subcellular and extracellular domain [11]. Perturbation theory, by which a solution to complex problem is approximated by solving a simpler but related problem, helps in this analysis [12].

The degrees of freedom for protein interaction in pathological states are enormous, with N → ∞. Further complexity is added by the time-space manifold of protein interaction, its dynamic nature and the large value of t, measured in years. Quantum field theory may help to comprehend the interacting fields of proteins, space-time, kinesis, and neurodegeneration.

The Liouville equation, which describes the evolution of density p of the system in phase space, may be written as:

$$i\partial p/\partial t=\hat{L}p$$

where $\hat{L}$ is known as the Liouville operator and i is the unit imaginary number defined by $i^{2}=-1$.

**Figure 3:**
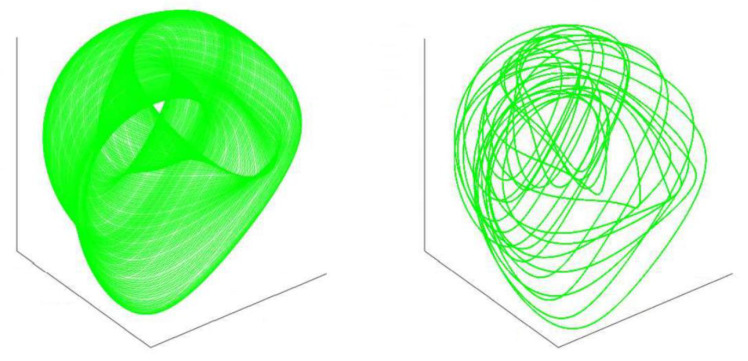
Idealized phase spaces representing all possible states in a neurodegenerative system, each phase state represented by one point of space-time, protein interaction, time, and movement (figure reproduced with permission from ref [13]. Copyright 2011 Elsevier).

Phase space = a space in which all possible states of a system are represented, with each possible state representing one unique point in the phase space (Figure 3, Phase spaces). The Liouville equation is a partial differential equation for the phase space probability distribution function.

Such spaces are of relevance to neurodegenerative processes where N→∞. In the context of interacting proteins in space and time, which result in a progressive neurodegenerative process, the evolution of the pathology is a result of the dynamics of correlations, influenced by the Liouville operator $\hat{L}$, which describes the time evolution of the phase space distribution function; such wave vectors allow the description of the evolution of molecular neurodegenerative pathophysiology as correlations derived from molecular interactions, i.e., an ensemble theory representing the probability distribution for the state of the system, and represented by the phase distribution function:

ρ

In large systems such as neurons, in which the molecular pathogenesis evolves, all properties (anatomical location, pH, *t*, phagosome function, etc.) exist in the limit N→∞ and result in the inexorable march of the neurodegenerative protein driven process and creates the mechanism of irreversibility. After time, this cascade leads to increasing degrees of freedom, and highly multiple and incoherent correlations, i.e., $PS_{ND}\;\to$ irreversibility as a result of continuous wave vectors as V→∞; the neurodegeneration progresses as a consequence of causality conditions as applied to *N*-body problems. The irreversibility of neurodegenerative molecular protein processes seems inevitable, determined by the nature of its physio-chemical properties, its non-equilibrium nature as the neuropathology progresses, and the role of entropy. The summation of all these factors causes the exponential transformation of the neurodegenerative process resulting in neuronal death, progressive neurological disability, and death of the subject as the pathology spreads through the intercellular space and into other brain cells.

It is posited that the theoretical framework developed so far on the nature of the interactions that influence the neurodegenerative process entropy is its driver. Living matter avoids the inert state of matter by drawing negative entropy from its environment (i.e., energy from food), being a neurone, the brain or the whole human [14]. Entropy is measurable and may be expressed by:

ENTROPY = K log D
K = Boltzmann constant (=3.2983 × 10^−24^ cal/°C)
D = A quantitative measure of the atomistic disorder of the body.

This molecular disorder is at the basis of neurodegeneration as the march of $PS_{ND}$ causes cellular and molecular disarray, increasing randomness of cellular and molecular machinery → breakdown of the neurone → loss of neurones as the molecular pathology spreads through the cell, extracellular space and the central nervous system:

→ Loss of brain structure (e.g., atrophy of the hippocampus in Alzheimer’s disease)
→ Generalized cerebral atrophy
→ Dementia
→ Inanition
→ Death and decay (thermodynamic equilibrium).

The human organism transforming from a low entropy state → high entropy state—in both intra- and extra-cellular space.

The natural history of neurodegenerative disorders such as Alzheimer’s disease, Parkinson’s disease, frontotemporal dementia, Prion diseases, and motor neuron disease are all progressive. As the neurodegenerative process begins, it has an inexorable march toward a state of high entropy equilibrium. Many attempts to hold this process with medications or monoclonal antibodies have not been successful, suggesting that the instigation of the neurodegenerative process is irreversible, propelled by the progression to increased entropy as dictated by the second law of thermodynamics.

**Figure 4:**
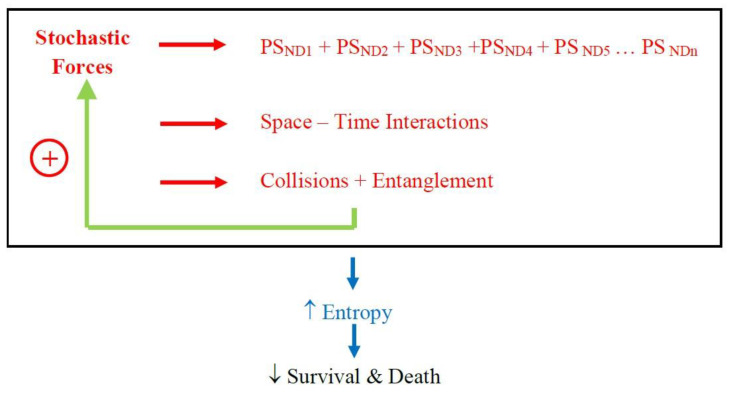
Stochastic mechanisms determine neurodegenerative protein sequences that interact in space-time, collide and become entangled with other proteins and structures → entropy increases and neurodegeneration ensues.

Support for the concepts presented here comes from recent clinic-pathological studies which show that patients with a clinical diagnosis of, say, Alzheimer’s disease might have, on neuropathological examination, multiple other protein deposits such as α-synuclein and transactive response DNA binding protein 43 [15,16]. These later proteins are associated with Parkinson’s disease, multiple system atrophy, frontotemporal dementia, and motor neurone disease. The presence of these other proteins is associated with a more severe clinical presentation and poorer prognosis. To explain, the stochastic forces determine multiple pathological proteins to be deposited in the brain; space-time interactions within cellular and extracellular compartments with molecular collision and entanglement with each other (and subcellular organelles) hastening the pathological process; with this disorder, entropy, increases exponentially, resulting in a more rapid clinical deterioration and poorer survival (Figure 4).

These considerations strongly suggest that prevention should be the optimum management strategy before the precise mechanisms enunciated in this paper are elucidated, including prevention of head injuries (even in children), a healthy diet, avoiding smoking and alcohol, good cardiovascular health—especially blood pressure management, regular physical exercise, cognitive and social engagement, and healthy sleep. Our findings submit that future research directed to understanding the stochastic basis (i.e., how random sequence changes at a peptide, RNA or DNA level → neurodegeneration) of molecular processes, the intra/extracellular kinetics of $PS_{ND}$ in three-dimensional space and countering entropy might yield new treatments, as our current treatments are symptomatic and do not change the natural history.

To this complexity must be added considerations as to the functional organization of the proteome and the intrinsic disorder of proteins. In the brain certain functions are integrated by networks [17] and that brain anatomical networks—the connectome—has topological properties [18]. Graph theory has helped to elucidate the operation of these networks in health and disease [19].

Protein-protein interaction networks control the functions of these large-array networks at a molecular level and are universally fundamental in all neuronal operations including synaptic function, neurogenesis, cell-cell interactions, autophagy and neuronal death [20]. Disturbed protein-protein interaction networks are elemental in neurodegeneration [21,22] This proteome can be measured using mass spectroscopy of biological fluids including cerebral spinal fluid and brain tissue [23]. As a consequence of these, disrupted protein-protein networks protein agglomeration leads to neuronal and glial cells distribution, neurones die, the neuropathology advances through the connectome, further neuronal loss occurs, the clinical state of the patient declines, the neuropathology proceeds through the interactome, and the neurodegeneration promotes advancing dementia, or muscular weakness as in the case of motor neuron disease, causing death.

**Figure 5:**
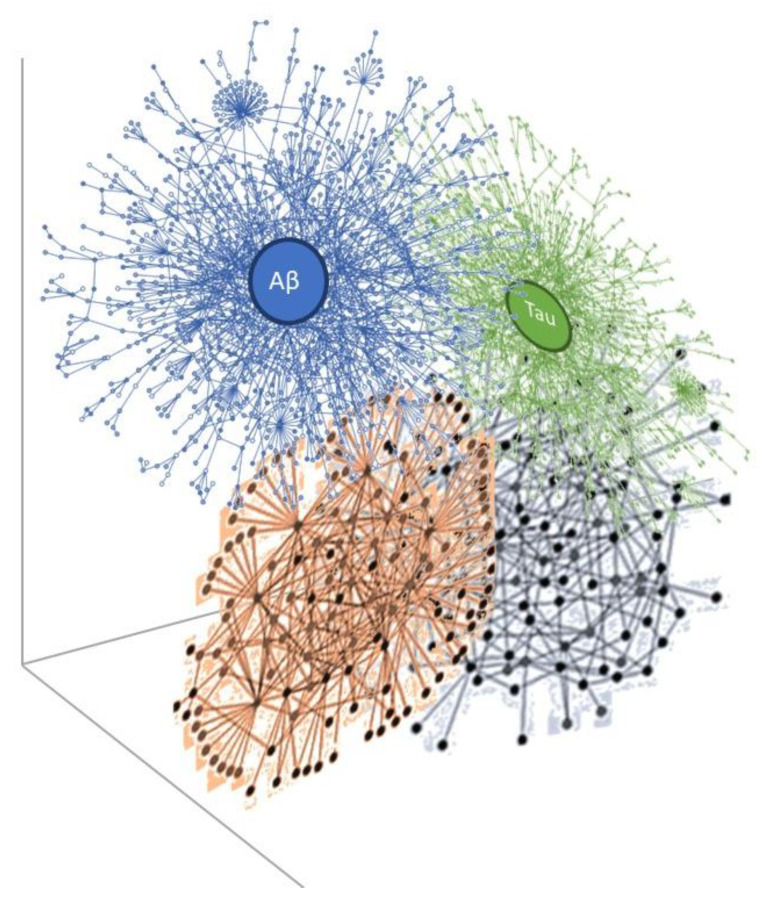
The interaction of protein networks and collisions in Alzheimer’s disease, as an example of neurodegeneration, with engagement of known proteins Aβ and tau, and yet-to-be-discovered proteins—unlabeled.

Furthermore, proteins have been shown to have intrinsic disorder which enables protein-protein networks to have “hub” connectivity, such that these proteins have the ability to bind and interact with a vast number of targets [24]. An analysis of experimental modeling in neurones of the ApoE ε4 allele, a risk factor for sporadic Alzheimer’s disease, shows a molecular impression of disrupted gene-protein-protein interaction networks [25]. We propose that in sporadic neurodegenerative disorders—and if we take Alzheimer’s disease as an example—the disturbed Aβ network interacts with the Tau network in three-dimensional space where they collide in space-time after a stochastic perturbation. This is conceptualized in Figure 5 where the disordered protein-protein interactive networks massively interact and contact other protein networks thought important in Alzheimer pathology—with other yet-to-be-discovered proteins contributing, possibly from the dark proteome [26] (these are shown unlabeled in Figure 5), i.e., the disrupted protein networks and protein network-protein network collisions compound the atomistic disorder promoting neurodegeneration (Figure 5).

Neurodegenerative disorders are characterized by neuronal loss and protein congregation. The pathological proteins project in the brain in a progressive manner, by seeding mechanisms and intercellular multiplication [27]. The popular processes believed to be operative in all sporadic neurodegenerative processes include neuroinflammation with microglial cells, the brain’s endogenous macrophages playing an important role which are activated by toxins, pathogens, peripheral inflammation, age, and chronic stress. Autophagy in which some proteins are broken down: there is macroautophagy—the principal pathway for removing damaged cellular organelles or proteins that are no longer needed; microautophagy—in which lysosomes evaginate the cytoplasm; chaperone—mediated autophagy is a proteolytic pathway that eradicates cytosolic proteins under certain conditions, molecular chaperones such as heat shock proteins stimulate this process. Macroautophagy has specific processes such as mitophagy (removal of mitochondria), lipophagy. and ribophagy. Oxidative stress is a process in which certain molecular species develop free radicals by autooxidation. Aging, toxins, and mitochondrial dysfunction generate reactive oxygen species (ROS), which results in cellular damage [28].

We posit that stochastic mechanisms provide the aberrant protein sequences that stimulate neuroinflammation, distort autophagy, and promote damaging oxidative stress. Furthermore, these abnormal protein sequences perturb the balance between phase separation and irreversible aggregation of proteins. Liquid-liquid phase separation (LLPS) of proteins and nucleic acids drives the formation of membraneless organelles, including neuronal stress granules in the cytoplasm and nucleoli, and paraspeckles in the nucleus [29,30]. These molecular condensates control subcellular chemical reactions and genetic flow of information from the nucleus. We posit that stochastically generated protein sequences disrupt these membraneless organelles and that randomly produced protein sequences, with prion-like realms and post-translational exchanges, distort the phase behavior of protein networks, resulting in disease [31].

**Figure 6:**
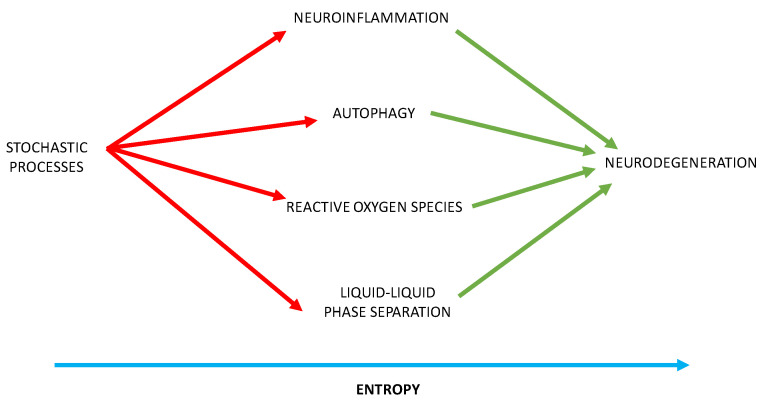
Stochastic processes and the mechanisms of neurodegeneration.

With these insights, our theory provides a mechanistic insight into the generation of neurodegenerative disorders, through which randomly generated sequences provoke neuroinflammation, autophagy, oxidative stress, and disrupt phase separation of proteins and nucleic acid causing the irreversible aggregation of proteins—with entropy providing the irreversible force resulting in inexorable neurodegeneration (Figure 6).

The limitations of this model are to hypothesize how stochastic variation in molecular sequences arises and what promotes their formation other than probability. This question raises the possibility of experimental verification of this model. Furthermore, in what cells—neurones, glia, and microglia—do these stochastic changes originate, and why do stochastic alterations favor certain anatomical locations; e.g., mesial temporal structures in Alzheimer’s disease and frontal lobes in frontotemporal dementia? The techniques of single cell proteomics offer an opportunity to examine the proteome of neurons and glial cells in tissue from brain bank collections to elucidate the cellular and anatomical diversity of stochastic protein variation in single cells from patients with Alzheimer’s disease, Parkinson’s disease, and other neurodegenerative conditions.

Additionally, DNA, RNA, and proteins could be extracted from brain tissue such that the total spectrum of sequence variation could be determined, and these sequences injected into experimental animals to assay relative pathogenicity—those sequences showing the greatest likelihood of promoting disease, allowing the development of molecular inhibitors

Organisms survive on the negative entropy extracted from food. Clinical trials using energy producing molecules such as glucose or treholose, given by infusions, might stay the progression of neurodegeneration.

Our findings support recent studies that suggest Alzheimer’s disease clinical symptomatology begins when tau aggregates exist in multiple brain regions; that is, inhibiting the initial stochastic sequence(s) that enhance neurodegeneration before propagation is likely to be an effective treatment and not preventing spread [32].

## 4. Conclusions

We submit that random changes in the sequences of DNA, RNA, or proteins is the fundamental pathological lesion in sporadic neurodegenerative disorders, and occurs in space-time vectors, with abnormal protein configurations propagating in intracellular and extracellular space leading to disrupted protein networks and cell death with entropy being the operator of relentless clinical deterioration and death. The merits of this concept are to understand that the majority of adult neurodegenerative disorders are sporadic and not related to gene mutations [33]. Our inquiries lead to novel therapeutic approaches into neurodegeneration such as space-time vectors of interacting protein networks and halting entropy’s relentless march. Stochasticity is considered a fundamental biological process important in our evolution and driven by entropy [34].

Finally, we propose that probability determines random sequence changes at a DNA, RNA, or protein level that result in pathogenic protein sequences, which misfold and cause neurodegeneration. This probability operates in anatomical space and may be represented by vectors in a phase space, influenced by time. These processes are determined by the second law of thermodynamics in which entropy is the driver, resulting in progressive neurodegeneration compounded by the atomistic disorder of protein network interactions.

Our clinical studies of young onset dementia provide clinical evidence for these stochastic processes [7].

## Data Availability

All data generated or analyzed during this study are included in this published article.
